# Three-Dimensional Constructive Interference in Steady State (3D CISS) Imaging and Clinical Applications in Brain Pathology

**DOI:** 10.3390/biomedicines10112997

**Published:** 2022-11-21

**Authors:** Marco Cavallaro, Alessandra Coglitore, Agostino Tessitore, Karol Galletta, Luciano Frosina, Antonino Cuffari, Roberta Ingrassia, Sarah Caroline Scarcella, Michele Caponnetto, Mirta Longo, Francesca Granata, Sergio Lucio Vinci, Enricomaria Mormina

**Affiliations:** Department of Biomedical Sciences and Morphological and Functional Imaging, University of Messina, Via Consolare Valeria 1, 98100 Messina, Italy

**Keywords:** brain pathology, magnetic resonance imaging (MRI), magnetic resonance cisternography (MRC), constructive interference in steady state (CISS)

## Abstract

Three-dimensional constructive interference in steady state (3D CISS) is a steady-state gradient-echo sequence in magnetic resonance imaging (MRI) that has been used in an increasing number of applications in the study of brain disease in recent years. Owing to the very high spatial resolution, the strong hyperintensity of the cerebrospinal fluid signal and the high contrast-to-noise ratio, 3D CISS can be employed in a wide range of scenarios, ranging from the traditional study of cranial nerves, the ventricular system, the subarachnoid cisterns and related pathology to more recently discussed applications, such as the fundamental role it can assume in the setting of acute ischemic stroke, vascular malformations, infections and several brain tumors. In this review, after briefly summarizing its fundamental physical principles, we examine in detail the various applications of 3D CISS in brain imaging, providing numerous representative cases, so as to help radiologists improve its use in imaging protocols in daily clinical practice.

## 1. Introduction

Constructive interference in steady state (CISS) is one of the most important sequences of the steady-state free precession (SSFP) family, and it is mainly used for the assessment of the central nervous system (CNS).

The SSFP technique was introduced in magnetic resonance imaging (MRI) more than half a century ago, and it was first described by Carr in 1958, who explained its basic physical principles concerning signal formation and properties [1]. Only at the end of the 20th century did the use of the sequence become popular.

In general, the term “SSFP” includes all steady-state sequences and their variants, which are named with different terms by MR manufacturers and divided into various types for different gradient switching patterns [2].

In this review, after briefly reviewing the main physical principles of this sequence, we aim to discuss the main advantages and limitations of the technique and to examine its most important clinical applications in brain imaging, so as to help radiologists optimize its use for the improved diagnosis of the main neuroradiological diseases in routinary practice.

### 1.1. CISS Properties

To explain how a steady-state free precession sequence is produced, some basic concepts concerning the interactions of the human body with an external magnetic field are to be introduced.

In a free state, protons of the human body are randomly oriented. When a patient is placed into the magnetic field of the MR scanner, the protons undergo a force that orients them according to a vector parallel to the external magnetic field (B0), generating a longitudinal magnetic field along the z-axis of the patient’s body, which is called longitudinal magnetization (LM) [3]. When a radiofrequency (RF) pulse is applied, the LM reverses in the transverse plane and produces a new entity called transverse magnetization (TM).

Generally, in conventional sequences, LM and TM vary in magnitude with subsequent excitations. In SSFP sequences, however, provided certain conditions are respected (a constant repetition time [TR] shorter than the T2 of the tissue, with very rapid RF pulses of the same flip angle), a dynamic equilibrium is reached, where the magnetization is constant from one repetition to the next (a “steady state” of magnetization, with constant LM and TM magnitudes). When this equilibrium is obtained, it is possible to collect two types of signals: free induction decay (FID), developing from the latest RF pulse, and spin echo (ECHO) signal, which occurs when the residual echo derived from the previous RF is refocused by the current RF wave. FID produces T1- and T2*-weighted (T1-w. and T2*-w.) images; ECHO generates strongly T2-weighted (T2-w.) images [4].

**Figure 1:**
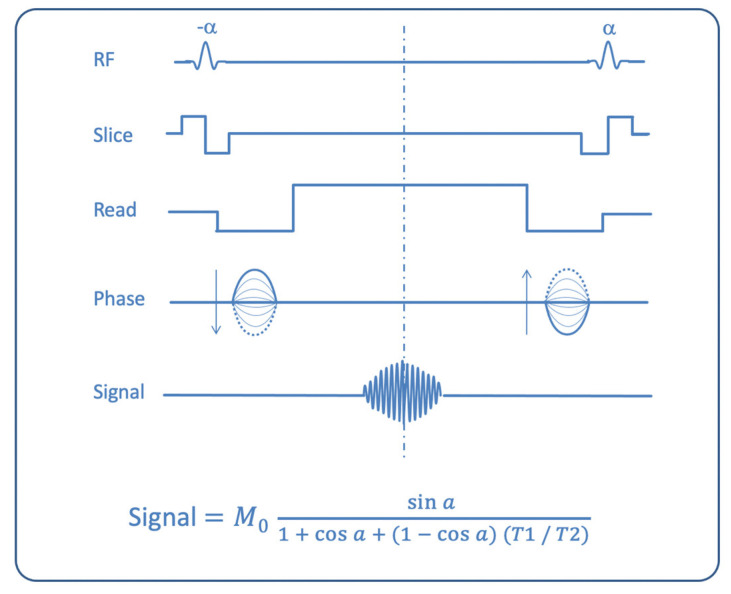
Scheme of a balanced Steady State Free Precession MR sequence, where balanced gradients along all three axes (slice-selection, phase-encoding, frequency-encoding) are used. In the formula (Huang et al., [5]), provided TR is much shorter than T1 and T2 of the tissue, it can be noted how signal intensity is directly related to the T2/T1 ratio at large flip angles. TR: repetition time; M0: magnetization at thermal equilibrium; α: flip angle.

The large family of steady-state gradient echo sequences can be classified into several groups: spoiled (or incoherent) steady-state sequences (where a spoiler gradient is used to dephase and cancel from the final signal the TM; sequence acronyms: FLASH, SPGR, T1-FFE), postexcitation refocused steady-state sequences (where only FID is used for signal formation; sequence acronyms: FISP, GRASS, FFE), preexcitation refocused steady-state sequences (where only ECHO is sampled; sequence acronyms: PSIF, SSFP, T2-FFE), and fully refocused steady-state sequences (where both FID and ECHO are used; sequence acronyms: true-FISP, FIESTA, balanced-FFE) [2,4,5]. This latter group is also called balanced SSFP (b-SSFP), because only in this group are the gradients in all three axes fully balanced, so that there is not a net gradient-induced dephasing of magnetization in each TR. In b-SSFP sequences, signal intensity is related to the T2/T1 ratio [6] (Figure 1).

This ratio is low for most of the solid organs and tissues in the human body, but is high in fat and, especially, fluids. This fact explains the low resolution in solid tissue differentiation (e.g., between gray matter and white matter in brain) on the one hand, and the elevated signal and excellent visualization of fluids—such as cerebrospinal fluid (CSF) and blood—on the other hand in b-SSFP sequences, which find therefore large application—in addition to brain MR imaging—in spinal, cardiac, abdominal (e.g., MR enterography) and fetal MR imaging.

CISS, as it is called on Siemens scanners, or FIESTA-C on GE (General Electric) systems, is a modified b-SSFP sequence which is obtained after a combination of two 3D b-SSFP acquisitions, with and without excitation pulse phase alternation, respectively. The aim is to reduce band artifacts, thus improving the image quality.

### 1.2. CISS Advantages and Limitations

When used in 3D mode, the CISS sequence has numerous advantages, such as the strong hyperintensity of the CSF signal, high signal-to-noise (SNR) and contrast-to-noise (CNR) ratios, absence of artifacts from magnetic susceptibility, high spatial resolution, better anatomical details of small structures, and topographical definition between the lesion and anatomical structures [7,8,9,10]. In addition, multiplanar (MPR) reconstructions (axial, coronal and sagittal) and curvilinear reconstructions can be performed [9,11].

Some limitations of the CISS sequence are the high cost, when compared to a conventional MRI scan, and the possibility of motion artifacts due to the relatively long image-acquisition times [6,12].

The thin contiguous sections and high in-plane resolution make it possible to depict minute structures. Any desired imaging plane can be obtained by the multiplanar reconstructive technique [1].

## 2. Literature Review

We searched the PubMed database in order to find clinical applications of the 3D CISS sequence in neuroradiological studies. The following terms were used: “CISS” and “brain mri”, “b_FFE” and “brain mri”, “DRIVE” and “mri brain”, “mri brain” and “FIESTA”, “CISS” and “neuroradiology”.

We found 138 articles for “ciss” and “mri brain”, 9 for “bFFE” and “mri brain”, 65 for “mri brain” and “FIESTA” and 60 for “CISS” and “neuroradiology”. We included only studies with CISS/DRIVE/b_FFE sequences and mri brain correlation, and also some relevant clinical, neurosurgical and histopathologic articles to show clinical applications of the CISS sequence in routinary neuroradiological studies. In all, only 88 studies were selected.

## 3. Clinical Applications

The 3D CISS sequence is used in addition to routine MRI sequences to study a wide range of pathologies, so as to obtain more detailed anatomic information. It is frequently used in the evaluation of numerous structures in the CNS, like cranial nerves (CNs), cisternal spaces, the cavernous sinus, the ventricular system, the spinal cord and related pathologies [13]. These structures are better displayed with this sequence [14].

### 3.1. Evaluation of Cranial Nerves

3D CISS, due to its cisternographic effect and anatomical details it can provide, is widely used for the assessment of CNs. In particular, the 3D CISS sequence can provide high-resolution images owing to the excellent contrast between CSF and solid structures, thus playing an important role in distinguishing between CNs, small vessels and CSF into the cerebellopontine angle (CPA) and other CSF spaces [15].

The first studies on this topic concerned the evaluation of the facial and vestibulocochlear nerves at the beginning of the 1990s [8]. Then several studies were conducted to study CNs and their relationship with adjacent structures [7,8,16].

#### 3.1.1. Olfactory Nerve (I)

3D CISS is an optimal sequence to study the anatomy of the olfactory nerve and olfactory bulb (OB); it shows with high resolution the olfactory tracts running through the CSF-filled olfactory grooves, as well as the cisternal segment of the nerve located below and between the gyrus rectus and the medial orbital gyrus [17].

The OB is the first cerebral olfactory tract that receives information from olfactory receptor neurons, and its volume is strictly related to afferent neural activities. In recent years, an increasing number of papers have reported a correlation between OB volumetric changes and several CNS disorders, such as Alzheimer’s and Parkinson’s disease, schizophrenia, depression, traumas, infections and chronic rhinosinusitis. It is also considered a complementary radiologic diagnostic tool and a prognostic evaluation factor for various olfactory disorders [18,19,20,21].

Burmeister et al., in a prospective study of 22 healthy volunteers, obtained excellent SNR and CNR values in a high-resolution 3T MRI protocol to study OB volume, with CISS sequence resulting the most suitable images for olfactory bulb volumetry. This is due to the high contrast resolution between the bright fluid signal and the very low signal intensity of the surrounding structures. The possible presence of artifacts and the poor contrast between gray and white matter represent, however, some limitations of this sequence [18].

#### 3.1.2. Optic Nerve (II) and Orbital Masses

With 3D CISS imaging, the different anatomic segments of the optic nerve (retinal, orbital, canalicular and prechiasmatic) can be easily studied and distinguished from the surrounding structures. 3D CISS thus greatly helps the study of the optic nerve and optic nerve sheath pathologies, showing with high-resolution details the relationship with adjacent structures. This is especially helpful in the evaluation of neoplastic diseases (like in optic nerve glioma and optic nerve sheath meningioma) in presurgical planning.

Another spectrum of diseases in which 3D CISS is particularly useful is represented by congenital and hereditary optic neuropathies, like optic nerve aplasia/hypoplasia and optic nerve coloboma. 3D CISS has proven to be able to demonstrate even very subtle colobomas that can be missed in other routinary sequences, and can be especially of help when fundus examination results are doubtful or hindered by other globe abnormalities [22].

**Figure 2:**
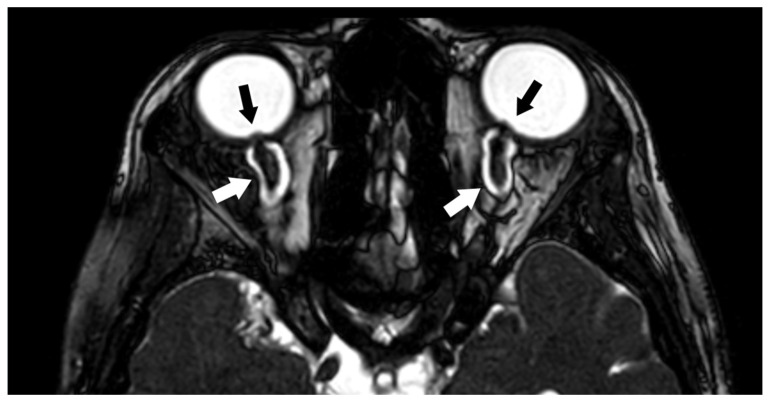
Axial three-dimensional constructive interference in steady-state (3D CISS) image showing in both sides posterior flattening of globes and protrusion of the optic nerve heads (black arrows), findings consistent with papilledema. Both optic nerves appear tortuous and the subarachnoid spaces surrounding them are enlarged (white arrows).

3D CISS is also important for the MR evaluation of papilledema. Papilledema is the result of increased intracranial pressure and can be caused by many different conditions, such as metabolic diseases, drugs, tumors, pseudotumor cerebri, hemorrhage, trauma, vascular and inflammatory processes. The 3D CISS sequence, due to the high contrast of liquid versus non-liquid structures, provides high contrast in the evaluation of the optic disc swelling, increased interstitial fluid in the optic nerve head, enlarged optic nerve sheath and elevation of the papilla into the globe (Figure 2) [23].

Owing to its high content in water, the ocular globe can be considered the ideal organ to study in high-resolution-MRI protocols, yielding high-quality images with elevated spatial resolutions utilizing the smallest possible field of view. The 3D CISS sequence is considered the best imaging modality to obtain the highest SNR, revealing the best contrast among the various ocular structures [24]. Thus, orbital structures other than the optic nerve, and orbital masses in general, can be optimally studied with 3D CISS, performed in addition to a standard MRI protocol.

CISS can help localize an orbital lesion to the compartment of origin (whether intraconal or extraconal, preseptal or postseptal), thus facilitating the diagnostic approach among the possible differentials. For instance, it can be very helpful in distinguishing retinoblastoma from rhabdomyosarcoma, two tumors more common in pediatric populations [17].

The sequence adequately shows the relationship between the orbital mass and the surrounding structures, especially with the optic nerve itself, while possibly showing additional neoplastic MRI features, like thickening and encasement of the nerve [7].

The high-resolution visualization of the relations between the mass and the surrounding structures is of great help for surgeons, both in preoperative planning and in the postoperative evaluation of possible residual tumors after a lesion resection [17].

#### 3.1.3. Ocular Motor Nerves (III–IV–VI)

**Figure 3:**
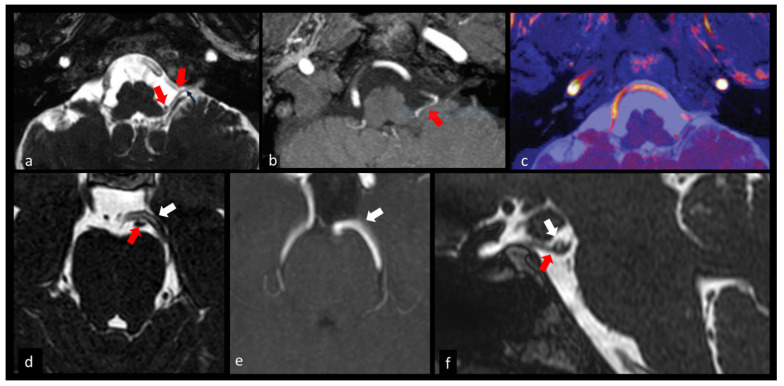
Neurovascular conflicts. (**a**–**c**) 71-year-old female with a 3-year history of intense pain localized in the pharynx and the posterior portion of the tongue. Axial 3D CISS (**a**) and 3D time-of-flight magnetic resonance angiography (3D TOF MRA) (**b**) show that the left posteroinferior cerebellar artery (red arrows) impacts the IX, X and XI cranial nerves at the root entry zone and along the intracisternal nerve tract. Postprocessed fused 3D CISS-TOF image (**c**) simultaneously displays the offending vessel (red color) and nerves (blue color). These images were released under CC license (with unrestricted use) (Alafaci et al. [25]). (**d**–**f**) Transient left third nerve palsy presumably caused by neurovascular compression. Axial (**d**) and sagittal (**f**) 3D CISS images show a close relationship between third nerve (red arrow) and posterior cerebral artery (white arrow) on the left side, with mild inferior displacement of the third nerve. 3D-TOF with maximum intensity projection (MIP) reconstructions (**e**) depicts the course of left posterior cerebral artery well. The patient spontaneously recovered from symptoms two days after the execution of magnetic resonance imaging (MRI).

In the setting of isolated or combined ocular motor nerve paresis, 3D-CISS has become a key sequence to be performed within the MRI protocol. It may explore with high accuracy the various segments of these complex-pathed nerves, showing possible sources of extrinsic compression causing palsy (Figure 3).

An example is given by the trochlear nerve (IV), the only CN arising from the posterior aspect of the brainstem, which is characterized by an exceptionally long course. Indeed, a focal nerve compression, especially in the tract adjacent to the cerebellar tentorium, can be easily overlooked in other sequences [17].

Sun et al. studied 17 patients with oculomotor palsy with a basic MRI protocol and with the addition of high-spatial-resolution 3D CISS images, showing the importance of such images in depicting with high image quality the relationship between the oculomotor nerve in the cisternal segment and its adjacent structures. They also showed a high correlation between MRI findings in 3D CISS images and surgical observations, demonstrating the importance of this sequence for the high-quality representation of anatomical details [26].

Park K-A et al. studied 127 young adult patients with isolated third, fourth and sixth cranial nerve palsies [26]. They found that the most common cause of palsy was represented by inflammatory diseases (21.3%), followed by microvascular causes (17.3%) and neoplasms, such as primary brain tumors—like meningiomas—involving the cavernous sinuses. High-resolution MRI was performed in all these patients, along with an additional 3D CISS sequence to visualize the cisternal segments of the ocular motor cranial nerves, which showed with high accuracy the sites of nerve compression and proved especially useful for diagnosing non-aneurysmal neurovascular compressions [26].

#### 3.1.4. Trigeminal Nerve (V)

The trigeminal nerve is the largest among the cranial nerves and the easiest to recognize in CISS imaging, as well as in other sequences.

A 3D CISS sequence is routinely part of the standard MRI protocol performed for the evaluation of patients with trigeminal neuralgia, in order to study the involved neurovascular structures and their relationships, as well as to potentially predict outcomes after microsurgical decompression [27].

The 3D CISS sequence is used for the identification of the site and degree of neurovascular conflict, recognizing both the artery and the vein responsible for typical and atypical trigeminal neuralgia, and for presurgical planning [6].

Compression and displacement of the trigeminal root entry zone by a vascular loop is considered the most common cause of trigeminal neuralgia. With a 3D CISS sequence, the anatomic details of the trigeminal ganglion, the nerve cisternal segment in the prepontine cistern and in Meckel’s cave in contrast with the CSF, and the thinning of the root entry zone caused by a vascular loop can be easily demonstrated [28].

In patients with refractory trigeminal neuralgia that underwent surgical intervention, a study of the anatomical structures of the nerve is performed as the initial screening procedure.

When analyzing 3D CISS images to search for possible neurovascular conflicts, it is mandatory to evaluate MR angiographic images as well, so as to clearly distinguish nerves from vascular structures, while possibly differentiating arteries from veins. In addition to a side-by-side comparison, it is also possible—after adequate postprocessing—to evaluate fused images, allowing a better discrimination of vascular and nervous structures. Miller et al., in 2008, studied 18 patients with unilateral trigeminal neuralgia. In their study, CISS images were fused to 3D TOF (3D Time of flight) angiographic and Gadolinium-enhanced 3D spoiled gradient recalled images, and an optimal correspondence between the preoperative imaging reconstructions and the intraoperative microscopic views was noted [29]. Held et al. showed the better sensitivity of multiplanar-reconstructed GE T1-w. images MPR-GE in recognizing the sensory and motor branches of the trigeminal nerve in comparison with reconstructions from a 3D CISS sequence, due to the isotropic voxels of the 3D MPR-GE dataset. However, 3D CISS was shown to be superior to a 3D MPR sequence in detecting both the trigeminal nerve and the Gasserian ganglion [29].

#### 3.1.5. Facial and Vestibulocochlear Nerves (VII–VIII Complex)

CISS imaging shows with high-quality resolution the courses of the facial and vestibulocochlear nerves: the root exit zone from the lower border of the pons, the crossing of the cerebellopontine angle cistern and the entrance through the porus acusticus into the internal auditory canal. Within the canal, the vestibulocochlear nerve splits into three components (the cochlear nerve and the superior and inferior vestibular nerves) which can be clearly depicted with CISS imaging.

One of the most important pathologies of these nerves is represented by schwannomas, which most frequently arise from the vestibular component of the vestibulocochlear nerve. Vestibular schwannomas are relatively common lesions and account for 75 to 85% of all CPA tumors (see below, paragraph 3.8: CPA lesions) [30]. Facial nerve schwannomas are far less encountered, although they represent the most common primary tumor of the facial nerve [31].

Neurovascular conflicts constitute another important pathologic condition involving the VII and VIII nerves. Specifically, a vascular compression of the root exit zone of the facial nerve can be responsible for hemifacial spasm (HFS). The root exit zone is considered the most vulnerable zone of the nerve and can be damaged by repeated pulsation of the responsible vessel. Other causes of HFS include tumors, demyelinating disorders and infections [32].

The 3D CISS sequence is strongly recommended in an MRI imaging protocol for the study of HFS, in combination with 3D TOF magnetic resonance angiography (MRA), to obtain fine anatomical details of the various structures with a good cisternographic effect. The result is an improvement of the positive rates and the overall accuracy for HFS diagnosis, compared with the 3D-TOF or 3D CISS technique alone [7].

A presurgical microvascular decompression was simulated by Granata et al., using virtual MRI techniques, such as 3D CISS and 3D-TOF images fusion and MRI cisternography, providing high spatial and contrast resolution of thin source images. In particular, the authors found an optimal correlation with surgical findings of the site of abnormal contact between the cranial nerves and the offending vessels, such as the superior cerebellar artery (SCA) and anterior inferior cerebellar artery (AICA), characterized by a “cross compression” with variable angle or a “sandwich compression” for the entrapment of the nerve between two vascular structures [33].

Painful tic convulsif is a rare pathological disorder caused by facial and trigeminal nerve compression by the SCA or the vertebral artery, clinically characterized by trigeminal neuralgia and ipsilateral HFS. Vertebrobasilar dolichoectasia is another uncommon cause, but it can present with ischemic events and compressive symptoms [34].

CISS imaging can be extremely useful in patients presenting with hearing impairment. In a study of 18 symptomatic patients with sensorineural hearing loss and/or vertigo, Held et al. demonstrated the correlation between high-resolution MRI findings and the presence of CPA masses or acute labyrinthitis. In particular, the contribution of the 3D CISS sequence was shown to be very important for providing the additional information of a filling defect in the CPA cistern, which corresponded to a circumscribed neoplastic mass, even in presence of very small tumors with a diameter of 2–3 mm [35]. A 3D CISS sequence can also be used to demonstrate the correlation between clinical symptoms of deafness and tinnitus ipsilaterally to the different types of internal auditory canal (IAC) vascular loops [36].

The 3D CISS sequence can be considered a complementary valid method for the early recognition of congenital VII or VIII CN aplasia/dysplasia.

In most studies in the literature, the morphometry of the facial and cochlear nerves was performed using high-resolution 3T MR to analyze auditory neuropathy spectrum disorders, like sensorineural hearing loss and facial palsy. Comparing a 3D CISS sequence performed at 3T and at 1.5 T MRI in the study of IAC structures, Garcia et al. demonstrated the superiority of 3T MR imaging for SNR and CNR, better delineation and anatomical details of cranial nerves, nerve branches and small vessels, with lower artifacts [37].

The morphometric study of the facial and cochlear nerves is becoming increasingly more important in clinical applications and management of Meniere’s disease, sensorineural hearing loss and facial palsy. Henneberger et al. demonstrated in 3D CISS images a significant enlargement in the volumetry of facial, cochlear and vestibular nerves bilaterally [38].

Peng L. et al. demonstrated a volumetric reduction of the cochlear nerve in patients with auditory neuropathy pathologies [39].

Ozdemir M. et al. considered the fundus as the best section where the cochlear and facial nerves can be identified. Various studies performed by some others reported the mean vertical diameters of these two nerves, varying between 1.10 ± 0.21 mm and 1.4 ± 0.21mm for the cochlear nerve and between 0.95 ± 0.21mm and 1.18 ± 0.17mm for the facial nerve [40].

#### 3.1.6. The Lower Cranial Nerves: Glossopharyngeal, Vagus, Accessory and Hypoglossal Nerves (IX–X–XI–XII)

Glossopharyngeal neuralgia is a rare disorder, frequently due to neurovascular compression, tumors, infarction, or dissection of the vertebral artery. A CISS sequence is used to reduce artifacts from CSF pulsation, obscure cisternal details, and provide better marginal delineation of the glossopharyngeal nerve in the cistern [41].

The cisternal segments of the IX, X and XI CNs are better visualized in vivo in combination with 3D CISS and 3D TOF sequences, which provide detailed information concerning the lengths of the cisternal segments of these nerves and the distances between the nerve root bundles and their dural meatus. The combined use of 3D CISS and 3D TOF imaging can help realize an accurate preoperative surgical planning with decreased perioperative morbidity and death (Figure 3) [25].

Also, the three parts of the CSF recess adjacent to the jugular foramen can be clearly visualized with the use of 3D CISS MR imaging techniques. In the craniocaudal sense, the three parts are:

- the recess for the cochlear aqueduct
- the recess for the glossopharyngeal nerve
- the recess for the vagus nerve and the accessory nerve [42].

The hypoglossal nerve is a pure motor nerve with a very thin structure; for this reason, it can be very difficult identify in standard MR images only. The contrast-enhanced 3D CISS sequence is considered superior to the T2-w. turbo spin echo (TSE) sequence for studying the entire cranial course of the nerve, its canalicular segments and its relationships to adjacent vessels, like the vertebral artery and postero-inferior-cerebellar artery, with high resolution, in contrast with CSF high contrast. Therefore, an enhanced 3D CISS sequence is used to evaluate nerve’s landmarks, like the nucleus of the 12th nerve at the posterior portion of the medulla oblongata, where it is responsible for a focal bulging of the floor of the fourth ventricle, designated as the “hypoglossal trigone” [43].

### 3.2. Virchow–Robin Spaces

Virchow–Robin spaces (VRS) are extensions of the subarachnoid spaces surrounding small arteries, arterioles, veins and venules, and they can be seen along the lenticulostriate arteries, surrounding the perforating medullary arteries in the convexities and in the midbrain. VRS are typically seen as well-defined oval, rounded, or tubular structures with regular margins, signal similar to CSF in all sequences, without any contrast enhancement; they can be considered enlarged when they measure more than 2 mm.

When they are atypical, they present in clusters or are markedly enlarged, causing mass effects, and they can be misinterpreted as other pathologic processes, like cystic neoplasms. Although benign in some situations, dilated VRS can present with hydrocephalus due to the compression of the cerebral aqueduct [8].

### 3.3. CSF Fistulae

CSF rhinorrhea can be present if there is a communication between the subarachnoid spaces and the sinonasal cavities. CT is mandatory to reveal bony defects, as for example, a defect of the cribriform plate, but a 3D CISS sequence can demonstrate the site of CSF leakage (the CSF fistula) by detecting a contiguous hyperintense CSF signal between the subarachnoid space and the sinonasal cavities [44].

CT cisternography is traditionally considered the gold standard technique for diagnosing CSF fistulae. However, CT cisternography is partially invasive, and the intrathecal administration of contrast material is often associated with headache and sometimes with signs of meningeal irritation, including seizures. Furthermore, the CSF leak has to be active to be detected with this technique; thus, patients with intermittent leaks can be underdiagnosed.

Numerous studies have demonstrated the effectiveness of 3D CISS imaging in showing the site of a CSF fistula by detecting a contiguous hyperintense CSF signal between the subarachnoid space and the sinonasal cavities [45].

**Figure 4:**
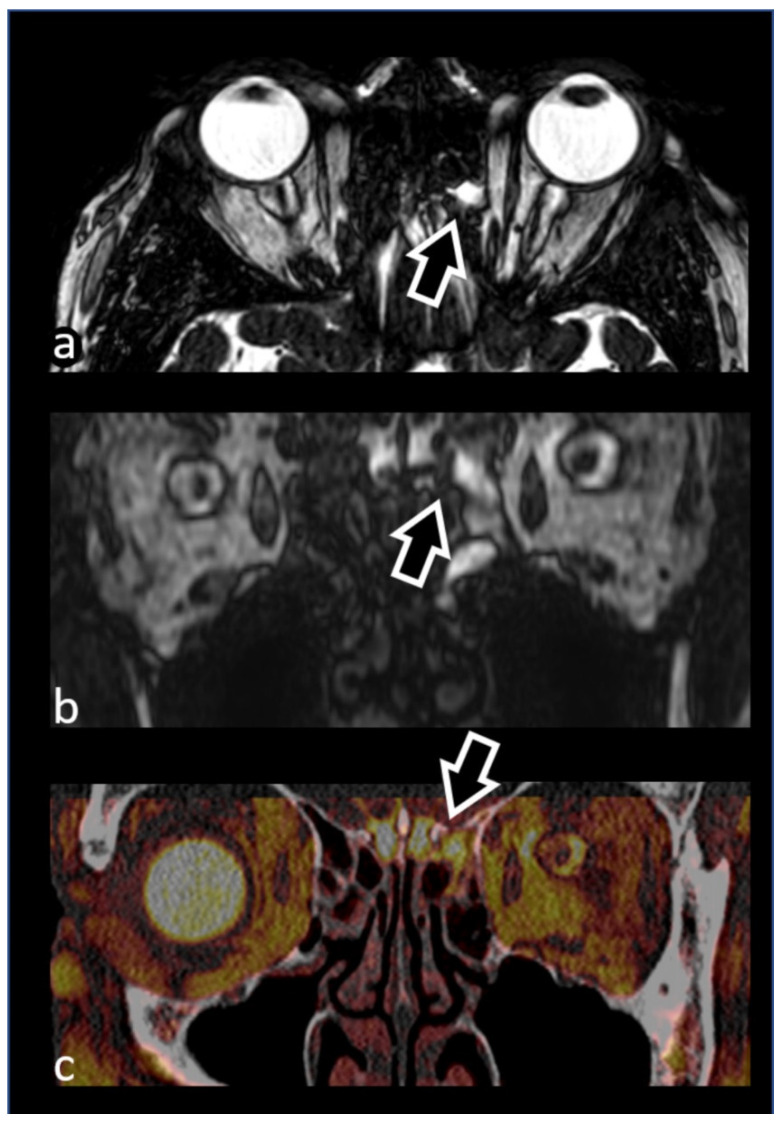
74-year-old female patient presenting with watery fluid leaking from her left nostril, suspected for cerebrospinal fluid (CSF) leakage. Axial (**a**) and coronal (**b**) 3D CISS images show a CSF-like linear hyperintensity extending from a subarachnoid space in the left frontal pole into an anterior ipsilateral ethmoid cell (arrows). At that level, computed tomography (CT) performed on the same day revealed a bony defect of the cribriform plate. These findings, well represented on postprocessed fused CT and MR-cisternographic images (**c**), were in keeping with a CSF fistula. A left partial ethmoidectomy was performed the following week, revealing a focal meningocele and confirming the CSF fistula. The meningocele was repaired, with a complete resolution of symptoms at one-month follow-up.

As the use of CT is paramount to reveal bony defects and to accurately assess the skull base and the paranasal bony structures, especially in preoperative planning, many centers propose to combine CT scans and MR-cisternographic images, with the possibility of obtaining postprocessed fused images, to detect the sites of both active and inactive CSF-leaks, reserving CT cisternography for equivocal cases [44,45] (Figure 4).

### 3.4. Cerebrovascular Pathology

Although other imaging modalities (digital subtraction angiography, computed tomography angiography, MRA) represent the gold standard for the study of brain vessel anatomy and pathology, the role that CISS imaging, due the very high spatial resolution it provides, can play in this spectrum of pathologies has been progressively recognized.

3D CISS can be very helpful in the therapeutic (interventional or surgical) planning of intracranial aneurysms, precisely depicting the aneurysmal sac and its relationship with adjacent structures. For instance, MRI with the use of b_SSFP and TOF-3D sequences proved to be useful in the study and classification of vertebrobasilar artery dissecting aneurysms, potentially helping to design individualized therapeutic strategies to optimize the treatment of aneurysms [46].

A specific type of aneurysms with which CISS-imaging can be especially useful is represented by internal carotid artery (ICA) aneurysms, located in the cavernous sinus (cavernous carotid aneurysms). These aneurysms, when symptomatic, can cause various neurological deficits, due to compression of the surrounding cranial nerves passing through the cavernous sinus itself (the abducens nerve) or its lateral walls (the oculomotor nerve, trochlear nerve, and ophthalmic and maxillary branches of the trigeminal nerve) [47]. As previously discussed, 3D CISS imaging is considered the main diagnostic tool to study cranial nerve segments and their relationships with surrounding structures.

For preoperative surgical programs, 3D CISS is useful in distinguishing paraclinoid from cavernous ICA aneurysms; this distinction is extremely important, as paraclinoid carotid aneurysms are associated with a major risk for subarachnoid hemorrhage, while cavernous sinus aneurysms usually require only follow-up in asymptomatic patients. Aneurysms can be classified on 3D CISS images on the basis of the relationship between the aneurysm and the superior wall of the cavernous sinus into: intradural, when they are surrounded by CSF; partially intradural, when a part of them is surrounded by CSF; extradural, when they are located into the cavernous sinus [48].

**Figure 5:**
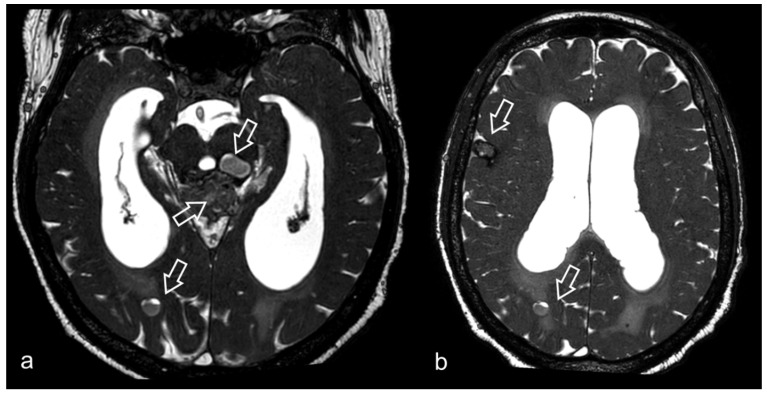
61-year-old male patient presenting with long-standing headache. MRI shows multiple supratentorial and infratentorial lesions, variable in size (some of the most prominent indicated by arrows), all characterized by marked blooming artifact in susceptibility weighted sequence (not shown). 3D CISS images (**a**,**b**) clearly show the composite structure of each lesion, made of multiple heterogeneous locules consistent with hemorrhage in different phases, surrounded by a hypointense rim due to hemosiderin. The most conspicuous lesion centered in midbrain tectum compresses the cerebral aqueduct, causing supratentorial hydrocephalus.

A CISS sequence can help better characterize cerebrovascular malformations, as well. In this context, it has been shown to be of value in the study of cavernous malformations (Figure 5). Especially when the cavernous malformation is localized in the brainstem, the sequence enables surgeons to know the exact distance between the cranial nerves in situ and the exact position of the malformation in the brainstem. 3D CISS is useful in identifying a safe entry zone into the brainstem, especially for pontine lesions where the nerve entry zone is located between the trigeminal and the facial or vestibulocochlear nerves. The high local resolution of 3D CISS enables surgeons to identify the peripheral rim of decreased signal intensity for hemosiderin deposits, indicative of venous anomalies of brainstem [49].

Sparing et al. described for the first time the importance of 3D CISS in studying thrombotic material located in extra- and intracerebral arteries. Due to the high contrast it produces between fluids and stationary tissues (like thrombotic materials) and its lesser susceptibility to flow patterns, it can play an important role in the identification of the site and nature of the stenosis or the occlusion of cerebral arteries [50].

**Figure 6:**
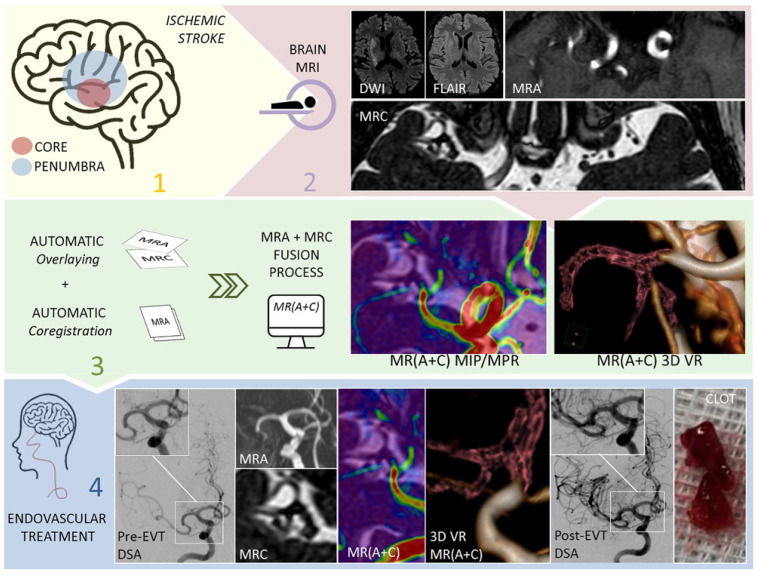
This figure shows the role that magnetic resonance cisternography (MRC) and magnetic resonance angiography (MRA), obtained with 3D CISS and 3D TOF, respectively, play in the diagnostic and therapeutic workflow in the setting of acute ischemic stroke (Mormina et al. [51,52]). This image was released under CC BY-NC-ND license (http://creativecommons.org/licenses/bync-nd/4.0/, accessed on 19 November 2022). 1 Clinical recognition of an acute ischemic stroke. 2 Brain MRI with the sequences diffusion-weighted imaging (DWI), fluid-attenuated inversion recovery (FLAIR), MRA and MRC. 3 Fusion process with the automatic overlaying and co-registration generates in a few seconds MR (A + C) fused images, with MIP, multiparametric (MPR) and volume-rendered (VR) reconstructions. 4 Endovascular treatment is performed on the patient affected by the acute ischemic stroke, with MR (A + C) serving as guidance.

3D CISS can play a substantial role in the setting of acute ischemic stroke (Figure 6). In candidates for endovascular treatment, an incomplete knowledge of the vascular anatomy beyond the site of the occluding thrombus can increase the procedure duration and the risk of complications or failure, especially in cases of complex vascular anatomy and vessel tortuosity. 3D CISS magnetic resonance cisternography (MRC), in combination with MRA, enables the depiction of vessels both before and beyond the site of obstruction, and it has been recently shown to be a precious guide for the interventional neuroradiologist, contributing to reduce the duration of the endovascular procedure [51,52].

Moya-moya disease is a cerebrovascular disease characterized by a progressive stenosis and occlusion in the terminal portion of the ICA, the anterior cerebral artery and middle cerebral artery, with markedly dilated perforating arteries (moya-moya vessels) to supply the collateral blood flow to the ischemic brain. Current studies in the literature have demonstrated the importance of the 3D CISS sequence, which can reveal a reduction not only of the luminal caliber, but also of the outer diameter of the carotid forks, as well as depicting the serial shrinkages of the involved arteries during disease progression [53].

Using 3D CISS, the outer diameter can be quantified in different vascular segments before and after the spontaneous disease progression. This sequence clearly shows the lower outer diameter of the involved arteries in parallel with progressive luminal stenosis; this phenomenon is considered the pathophysiologic mechanisms underlying the development of moya-moya disease [54].

In the pediatric population, 3D CISS images can be used to show the presence of intracranial venous anomalies. In particular, the vertical embryonic positioning of the straight sinus is a rare venous developmental anomaly, probably caused by an incomplete resorption of a large meningoencephalocele during fetal life or the result of persistence of neural crest remnants. Images clearly show a CSF space that contains an ascending flow void from the inferior sagittal sinus and extends superiorly to the base of the subscalp lesion. The identification of an atretic cephalocele can lead to other additional intracranial anomalies and correlate with variable clinical outcomes, like marked cerebral dysplasia, severe retardation and death [55].

The high-resolution of 3D CISS in depicting vessel anatomy can represent a possible non-invasive tool for studying very small arteries and veins that are difficult to evaluate in classic protocols, such as the ethmoidal artery, whose distance to the extraocular muscles in blowout fractures and course and length in candidates for functional endoscopic sinus surgery can help prevent surgical and postsurgical complications [56]. Future studies are needed to evaluate the effective role of 3D CISS in this setting.

### 3.5. Hydrocephalus, Ventricular System and Subarachnoid Cisterns

In most MR imaging protocols, 3D CISS is performed in patients with hydrocephalus, for the evaluation of anatomical details and the assessment of CSF pathways [57].

Obstructive hydrocephalus is a type of non-communicating hydrocephalus caused by obstruction of CSF pathways at different levels.

At the level of the foramina of Monro, a colloid cyst can represent a cause of hydrocephalus. Other common causes can be brain tumors and inflammatory diseases. Inflammatory septa, membranes, neurocysticercosis, arachnoid cysts, lesions in the pineal gland and tectal gliomas are the most common causes of aqueduct obstruction. The most common causes of compression at the level of the foramen magnum are congenital malformations of the skull base and of the cranio-cervical junction, like Chiari malformations [58].

**Figure 7:**
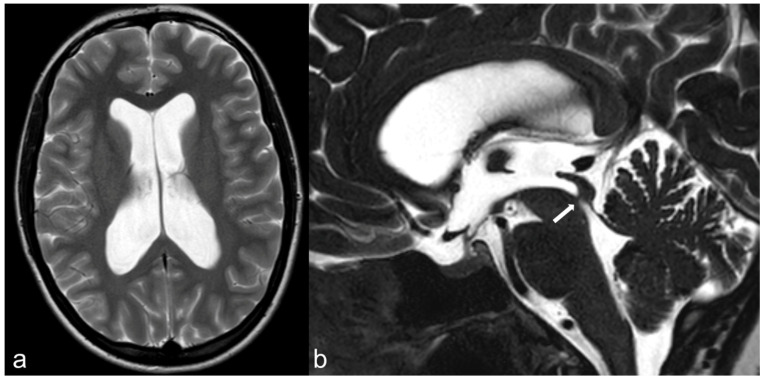
26-year-old female patient presenting with long-standing headache, unresponsive to medical therapy. MRI reveals obstructive supratentorial hydrocephalus. (**a**) Axial T2-weighted (T2-w.) turbo spin echo (TSE) image shows enlargement of the lateral ventricles. No signs of transependymal edema are apparent. (**b**). 3D CISS depicts a subtle hypointense membrane (arrow) located at the level of the cerebral aqueduct. Findings are consistent with aqueductal web. The patient subsequently underwent endoscopic third ventriculostomy.

Among the different causes of hydrocephalus, CISS imaging is especially effective in demonstrating the presence of membranes inside the ventricular system, a condition which is often difficult to show on conventional imaging sequences. The presence of aqueductal webs or membranes is the most common condition responsible for congenital aqueductal stenosis, which is in turn the most frequent localization of congenital obstructive hydrocephalus (Figure 7). Comparing the efficacy of a phase-contrast MRI and a 3D-CISS sequence for the evaluation of aqueductal stenosis, Algin et al. proposed a grading score for aqueductal stenosis on the basis of the degree of obstruction on CISS images, comprising a “grade 1 stenosis”, with a narrowed but still patent aqueductal lumen, and a “grade 2 stenosis”, where the aqueductal lumen appears occluded or a stenosis–prestenotic dilatation secondary to the web is visible. 3D CISS imaging is considered particularly useful for the anatomical information regarding aqueduct morphology it may provide, which can be especially helpful before surgery or endoscopic third ventriculostomy (ETV) [59].

Intraventricular choroid plexus cysts can be responsible for acute hydrocephalus, and the diagnosis may not be always simple, as the presence of thin walls around them and their content similar to CSF may cause them to go undetected in routine CT studies. Clinical presentation depends on cyst volume, and it can be severe, with acute neurological deterioration, headache and obstructive hydrocephalus, requiring urgent surgery. In the literature, the superiority of b-FFE 3D sequences compared to fast SE-T2-w. sequences has been reported, due to higher definition images, lower artifacts and shorter acquisition time [15,60].

CISS sequences can be employed to study the anatomy of the third ventricle, in the preoperative planning of ETV, as a complementary sequence to routinary MRI protocols, suggesting the best site for successful fenestration in neuroendoscopic surgery, and showing postoperative findings like the status of the stoma, surgical defects and the presence of membranes or adhesions [61]. It is also important for the evaluation of postoperative hydrocephalus [62]. Furthermore, MRI with CISS images may disclose unexpected abnormalities, thus changing the strategy of treatment in cases of atypical hydrocephalus and providing essential information for surgical planning [58]. In this context, 3D CISS imaging has proved to be very effective in the representation of the Liliequist mebrane. The Liliequist membrane is a very thin arachnoid membrane that arises from the dorsum sellae to the anterior edge of the mamillary bodies. Incomplete opening of the Liliequist membrane has been claimed as a possible factor responsible for failure of ETV [63].

The 3D CISS sequence can be used for the delineation of the entire anatomic course of the Liliequist membrane, showing its single segments (sellar, diencephalic and mesencephalic), with the sellar segment being the best delineated segment [64]. Thus, the study of this membrane can be important both for a more accurate preoperative planning and for the postoperative evaluation of ETV, and it could be important for a better understanding of some cases of suprasellar arachnoid cysts and perimesencephalic hemorrhages [65].

Intraventricular hemorrhage is a common pathologic condition, secondary to various vascular diseases like hypertension, arteriovenous malformations, angiomas, aneurysms and moya-moya disease; idiopathic intraventricular aneurysms are rare. 3D CISS sequences and DSA can rule out other causes of intraventricular bleeding, like the presence of choroidal artery aneurysms associated with the parent vessel [66].

**Figure 8:**
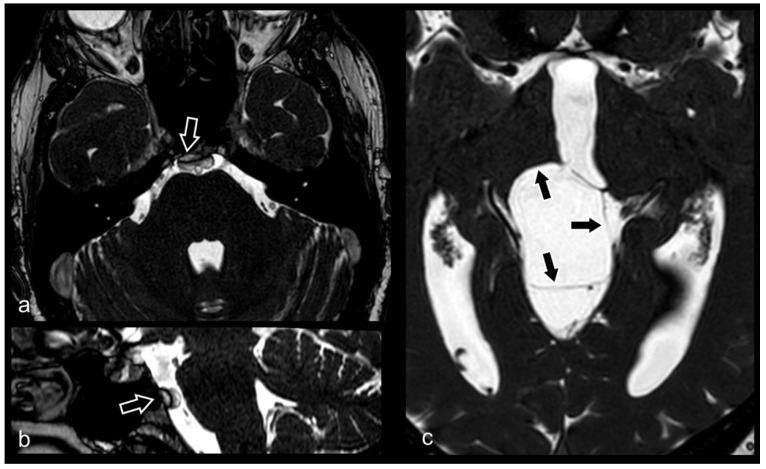
(**a**,**b**) Focal extra-axial CSF-like lesion (arrow) located in the prepontine cistern. The lesion is strictly adherent to, and partially extends into, the clivus, and abuts the basilar artery, without signs of mass effect. Location and signal characteristics are in keeping with ecchordosis physaliphora. (**c**) Axial 3D CISS image shows a quadrigeminal cistern arachnoid cyst (cyst walls indicated by arrows) causing supratentorial hydrocephalus.

3D CISS is extremely helpful in the study of the cisternal spaces and related pathologies, ranging from infections to malignancies and inflammatory diseases. Indeed, numerous pathologies—like bacterial (especially tuberculosis) or carcinomatous meningitis, fungal infections, neurosarcoidosis and many others—are characterized by a typical involvement of cranial basal cisterns. These conditions are usually studied on contrast-enhanced images, which are often sufficient for diagnosis, especially in cases of diffuse disease. However, in cases of subtle or initial disease, it is possible that basal infiltrates or infectious/inflammatory granulomas can be overlooked on conventional images, especially if they are little, are isointense to CSF or do not present contrast enhancement [7]. In these cases, 3D-CISS can optimally show the pathologic components in the cisternal spaces in contrast with the surrounding CSF. An example of pathology typically centered in the prepontine cistern is represented by two entities arising from notochord remnants: retroclival cordoma and ecchordosis physaliphora (Figure 8). Indeed, these lesions are increasingly diagnosed—usually as incidental findings—due to the diffusion of the 3D CISS sequence, because, owing to their high T2 signal and subtle margins, they can be easily overlooked in routine MRI protocols, especially if small and asymptomatic.

Common lesions frequently located within the cisternal spaces are arachnoid cysts (Figure 8). Usually asymptomatic, they can however exert signs of mass effect and even hydrocephalus when excessively large.

**Figure 9:**
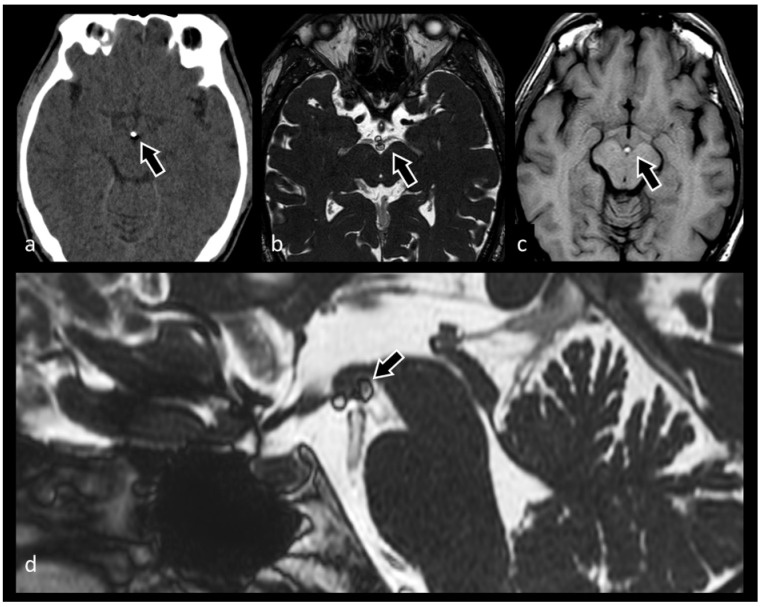
Axial brain CT (**a**) shows two incidentally-discovered focal calcified formations (arrow) located in the interpeduncular cistern, indissociable from the basilar artery. Axial and sagittal 3D CISS images (**b**,**d**) characterize the formations (arrow) and their relationship with the basilar artery well, and show the calcifications are mainly located at the periphery of the lesions, appearing as subtle hypointense rims. MRI also reveals the lesions (arrow) present a lipidic core, which appears hyperintense on spin-echo T1-w. sequence (**c**) and shows signal dropout on fat-suppressed images (not shown). Findings were consistent with osteolipomas.

Eventually, 3D CISS can demonstrate with precise details small and benign incidental lesions, such as osteolipomas, which appear otherwise non-specific in other sequences or imaging modalities (Figure 9).

### 3.6. Neurocysticercosis

Neurocysticercosis is the most common helminthic infection of the CNS, and it can present with atypical forms, such as intraventricular, subarachnoid, spinal, orbital and intra-parenchymatous forms. 3D CISS is the best non-invasive method for evaluating patients with neurocysticercosis, especially if the cestode is located in the ventricular system or subarachnoid space, or when the scolex is situated inside the cysticercus cyst, antigenically non-visible, without enhancement or edema [14,67].

Patients can develop hydrocephalus, because small focal lesions of the sellar region may be responsible for arachnoiditis and obstruction. These lesions can be misdiagnosed as a hypophyseal adenoma with cystic degeneration, craniopharyngioma, arachnoid cyst, partially calcified giant carotid aneurysm or epidermoid tumor. FLAIR and 3D CISS sequences are the best non-invasive advanced MRI techniques to study lesions in these locations. The 3D CISS sequence is considered a great tool for the evaluation of intraventricular and subarachnoid cysticercal cysts, showing the cystic wall, cystic fluid and scolex [14]. Spin echo (SE) T1-w. imaging and TSE T2-w. imaging have been shown to be less sensitive than the 3D CISS technique for the diagnosis of intraventricular cysticercal cysts [68].

### 3.7. Evaluation of Brain Tumors

**Figure 10:**
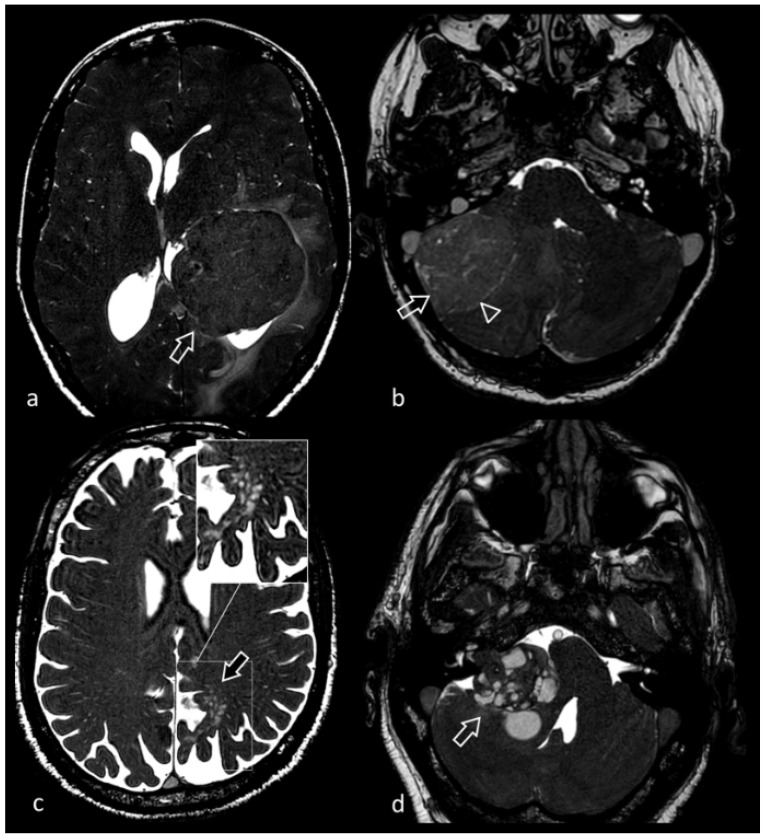
(**a**) Axial 3D CISS image shows a large mass (arrow) in the left lateral ventricle. 3D CISS accurately depicts the endoventricular location of the mass and helps characterize its relationship with the surrounding brain tissue. Histological findings were in keeping with intraventricular fibrous meningioma. (**b**) 32-year-old female patient with a large lesion (arrow) in the posterior fossa, initially suspected for a cerebellar neoplasm. 3D CISS optimally demonstrates a subtle hyperintense rim between the mass and the cerebellar hemisphere (CSF cleft sign, arrowhead), suggestive for the extra-axial location of the tumor. After brain surgery, final diagnosis was extra-axial desmoplastic medulloblastoma of the right cerebellopontine angle. (**c**) Incidental subcortical and cortical lesion (arrow) in the left parietal lobe, hyperintense in T2-TSE and FLAIR sequences, devoid of contrast enhancement or diffusion restriction, and without signs of mass effect. 3D CISS shows with high definition that the lesion is mainly made of a cluster of multiple well-defined «bubbles». Findings were consistent with a multinodular and vacuolating neuronal tumor. (**d**) 62-year-old woman. Axial 3D CISS image shows a well-defined lesion, with solid and cystic components, in the right cerebellopontine angle that partially extends into the internal acoustic canal and causes compression of the antero-lateral portion of the pons, which was later diagnosed as vestibular schwannoma histopathologically.

3D CISS, due to the cisternographic effect and the high resolution, is the ideal sequence to precisely delineate the margins of tumors located in the ventricular system (Figure 10a). In this case, reconstruction images can easily depict the relationship between the lesion and outlets flow, such as the foramen of Monro, mesencephalic aqueduct and basal cisterns. In the periaqueductal region, 3D CISS proved to be important in the correct diagnosis of tumor origin (tectum or aqueduct). This is paramount, as treatment strategies for tectal glioma and pure aqueductal tumors are different: tectal glioma usually requires observation, but if a pure aqueductal tumor is suspected and shows hydrocephalus, a biopsy should be performed at the same time as ETV [69].

**Figure 11:**
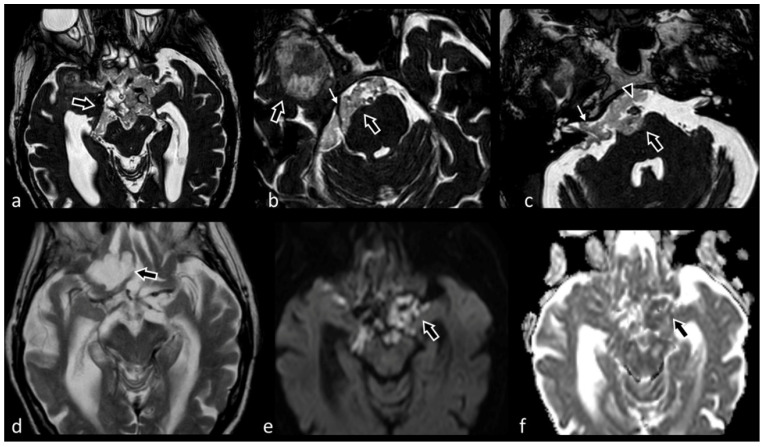
Voluminous extra-axial lesion centered in the suprasellar cistern, diffusively extending into the contiguous cisternal spaces as well as into Sylvian and choroid fissures bilaterally; inferiorly, the mass also runs along the prepontine cistern, the Meckel’s cave and the internal auditory canal (small white arrow in (**c**). The lesion appears isointense to CSF in T2-TSE (**d**), markedly hyperintense on DWI (**e**) and partially and lightly hypointense on ADC maps (**f**). 3D CISS (**a**–**c**) is superior compared to the other sequences in demonstrating the internal texture of the lesions, its margins as well as its relation with surrounding structures (e.g., fifth cranial nerve, small white arrow in (**b**), and basilar artery, arrowhead in (**c**), which are encased but not dislocated by the mass). These features, alongside the characteristic hyperintensity in DWI, are highly suggestive for an epidermoid cyst.

3D CISS also represents the best sequence for distinguishing intra-axial from extra-axial tumors and shows with high accuracy the CSF cleft, useful in characterizing a lesion as extra-axial (Figure 10b and Figure 11). The recognition of adjacent nerves and vessels it provides is useful, as well, in defining the exact location of the tumor [7].

Apart from extent, margin and relationship with surrounding structures, 3D CISS also depicts with high accuracy the intralesional cystic components delineated by a thin wall, and can help in the differential diagnosis of lesions such as dysembryoplastic neuroepithial tumours, ganglioglioma, and multinodular and vacuolating neuronal tumors (Figure 10c,d) from other apparently similar pathologies [70].

### 3.8. Cerebellopontine Angle Lesions

The cerebellopontine angle (CPA) is a triangular cisternal space located in the posterior cranial fossa between the tentorium superiorly, the pons posteromedially and the temporal bone laterally. It is crossed by the V, VI, VII and VIII CN and by the AICA.

Benign and malignant lesions may spread from the CPA along the basal cisterns, and they can be detected with 3D CISS sequences. A great variety of lesions that primarily occupy the CPA cistern or that extend into the CPA from adjacent regions exists, presenting with sensorineural hearing loss, tinnitus, dizziness or non-specific symptoms [71]. The majority of the lesions in the CPA are benign, slow-growing tumors, with low risk for malignant transformation.

Vestibular schwannomas represent the most common lesion encountered in the CPA, accounting for 75 to 85% of all CPA tumors (Figure 10d) [30]. More rarely, schwannomas of the CPA can arise from other CNs. Trigeminal schwannomas, which account for around 0.07–0.3% of all intracranial tumors, constitute the second most common intracranial schwannomas and can develop from one of the three branches of the nerve (more rarely), from the trigeminal ganglion in Meckel’s cave or from the cisternal segment of the nerve (in the prepontine or CPA cistern) [72].

Even less frequently, schwannomas can arise from the facial, the glossopharyngeal, the vagus and sometimes even the accessory cranial nerves. It represents the most common primary tumor of the facial nerve.

Vestibular schwannomas can be sporadic unilateral tumors or can be associated with neurofibromatosis type 2, where they tend to be bilateral.

Nakai et al. evaluated 82 patients treated for vestibular schwannoma, assessing preoperative contrast-enhanced 3D T1 sequence and 3D CISS images. Comparing preoperative MRI findings with intraoperative findings, they found a correlation of 74 % between nerve location and intraoperative findings [73]. 3D CISS images accurately demonstrate the topographical relationship between the tumor and the facial and vestibulocochlear nerves, which is especially useful for the improvement of microsurgery and endoscopic surgery in the internal acoustic canal [74].

Meningiomas are the second most common CPA tumors and originate from the proliferation of arachnoid meningothelial cells of the dura of petrous temporal bone or internal auditory canal. The majority of meningiomas are benign, slow-growing (grade I) tumors, and the most common subtypes are meningothelial, fibroblastic and transitional [75]. In the preoperatory planning of safe resections of skull base meningiomas, high-resolution CSF-sensitive sequences are considered the best imaging method to identify cranial nerves within cisternal spaces, among conventional MRI sequences [76].

Epidermoids are ectodermal tumors that arise from congenital misplacement of ectodermal cells during the closure of the neural tube. They are located in the subarachnoid spaces and are generally studied with CISS and FLAIR sequences, which provide a better contrast between tumors with less artifactual interference. In CISS sequences, it is possible to recognize the exact tumor extent of even small lesions. Epidermoids appear hypointense relative to CSF and they are filled with cholesterol crystals and keratin, with variable signal observed on the CISS images, depending on the substances contained within [77] (Figure 11).

Arachnoid cysts are CSF-filled cysts that arise from the splitting of embryonic arachnoid membranes, occasionally found in cisternal spaces. A 3D CISS sequence is strongly recommended for the preoperative planning of neuro-endoscopic surgery in the evaluation of symptomatic and growing arachnoid cysts. Postoperative controls with this sequence may also demonstrate the difference between a recurrent arachnoid cyst and a postoperative cavity [78].

Lipomas are fatty tumors that can on rare occasions be located at the CPA, representing around 0.1% of all tumors in the CPA [79].

Nerves and blood vessels situated in this region can cross through the tumor generally without showing signs of displacement. They can be associated with intravestibular lipomas and vestibulocochlear malformations; thus, careful observation for associated abnormalities and integration with CT is required, especially in patients presenting with sensorineural hearing loss [80].

As previously discussed, 3D CISS is extremely useful for evaluating pathologies affecting CNs crossing the CPA and neurovascular conflicts. 3D CISS can also detect other lesions which rarely involve the CPA (like metastatic lesions, glomus jugulare, primary brain tumors, etc.), that can be missed on other MRI sequences. For instance, in a case report described by Yousry I. et al., 3D CISS images, in combination with 3D MP-RAGE, provided the identification of an intra-axial tumor, an adult pilocytic astrocytoma, that grew up exophytically from the brainstem to the right cerebellopontine angle. A precise relationship between the tumor and CN VIII was documented, as well as the encasement of CNs IX–XII, providing an optimal preoperative planning [81].

### 3.9. Hypothalamic–Pituitary Disorders

3D CISS can be used as a complementary sequence in addition to conventional SE image in the pediatric population to study small hypothalamic hamartomas surrounded by CSF.

The 3D CISS sequence can show with greater anatomical details even small hypothalamic hamartomas in comparison with SE T1- and T2-weighted images, due to very thin sections without intersection gaps. A 3D CISS sequence can provide a CNR three times higher than SE sequences, with resulting better differentiation of parenchyma surrounded by CSF from the structures contained in the CSF. In addition, artifacts from CSF flow are fewer in 3D CISS images [82].

In the suspicion of pathologies causing pituitary stalk (pituitary stalk) dysfunction—including congenital hypopituitarism, genetic defects, tumors, Langerhans cell histiocytosis, inflammatory, autoimmune and infectious diseases—MRI provides an optimal amount of information, especially after administration of gadolinium. Godano et al. compared the diagnostic information obtained with 3D-CISS and T1-weighted sequences in patients with pituitary region abnormalities. They considered the 3D-CISS sequence more reliable than the T1 precontrast sequence in the evaluation of precise measurements of the PS and in the identification of PS abnormalities, associated with similar or even additional information when compared to postcontrast T1 in the visibility of PS and PS size [83].

### 3.10. Cranio-Cervical Malformations (Chiari I)

Dawes et al., in a study of 88 patients with Chiari I malformation, using a cine bFFE technique, studied the abnormal CSF flow dynamics, in particular the relationship between Valsalva headache and the altered tonsillar motion at the cranio-cervical junction during the cardiac cycle. This study demonstrated both displacement and tissue strain, with Valsalva maneuvers being responsible for only transient changes in intracranial CSF pressure [84]

### 3.11. Evaluation of Internal Auditory Canal and Inner Ear Structures

3D CISS sequences at 3T are considered state-of-the-art imaging methods for MR cisternography for the assessment of the anatomic structures of the IAC and inner ear. Byun et al. showed that these sequences can provide a good quality of images, though in the evaluation of the basal turn of the cochlea, vestibule and all semicircular canals they were limited by the presence of magnetic susceptibility artifacts [85].

Patients with sensorineural hearing loss, candidate for cochlear implants, are studied with high-resolution CT imaging for the temporal bone and with CISS sequences for the evaluation of morphology of anatomic structures of the IAC. In particular, Kim et al. showed the usefulness of 3D CISS technique with volume rendered reconstructions to diagnose congenital anomalies of the semicircular canals and common crus aplasia, one of the most common congenital cause of sensorineural hearing loss [86].

### 3.12. Neonatal and Fetal MRI

**Figure 12:**
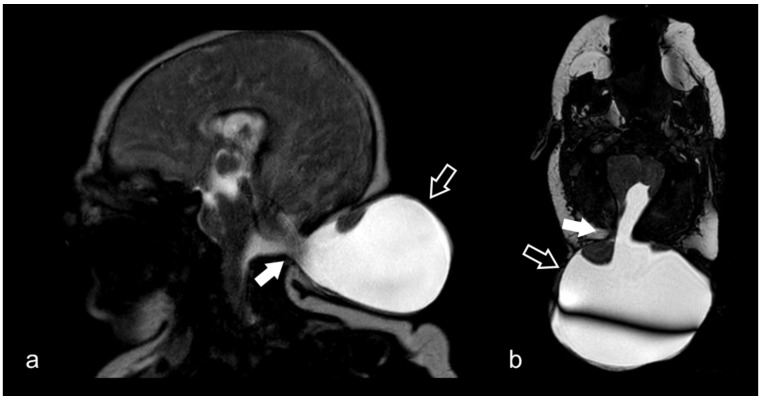
Sagittal (**a**) and axial CISS (**b**) images showing occipital meningoencephalocele (black arrows). Voluminous herniation of meningeal membranes, CSF and partially of brain tissue through a wide skull defect in the occipital bone (white arrows). Hypoplasia of cerebellum and dysmorphic brainstem can also be noted.

3D CISS is often useful for the definition and correct classification of several congenital brain malformations. For instance, it can show with high accuracy malformations of the posterior cranial fossa, as well as supratentorial and infratentorial meningo/meningoencephaloceles (Figure 12).

In preterm infants, hydrocephalus can frequently be observed, generally a consequence of intraventricular haemorrhage. Fadzli et al. reported a case of quadriventricular hydrocephalus caused by foraminal septa requiring surgical intervention. A 3D-CISS sequence is strongly recommended to demonstrate the presence of septa at the level of the fourth ventricular foramina, as well as to help with presurgical planning and to achieve a successful endoscopic third ventriculostomy, due to the high anatomical resolution it can provide [87].

Persisting embryonal infundibular recess (PEIR) is a rare anomaly of embryogenesis, characterized by the presence of a cyst in an enlarged sella, with the anterior and posterior pituitary lobes displaced superiorly. The 3D CISS sequence is useful for detecting the communication tract between the floor of the third ventricle and the cyst of PEIR, thus helping differentiate this condition from other lesions, like empty sella, cystic adenoma, Rathke’s cleft cyst, craniopharyngioma and arachnoid cysts [88].

## 4. Conclusions

3D CISS sequence provides superior detailed information about a wide range of brain pathologies. Its cisternographic effect and high-resolution anatomical details make this sequence suitable for imaging and problem solving in numerous neuroradiological scenarios, often providing precious information that helps optimize diagnosis and patients’ healthcare.

## Abbreviations

b-SSFP	balanced steady-state free precession
CISS	constructive interference in steady state
CN	cranial nerve
CNR	contrast-to-noise ratio
CPA	cerebellopontine angle
CSF	cerebrospinal fluid
ETV	endoscopic third ventriculostomy
IAC	internal auditory canal
MRA	magnetic resonance angiography
MRC	magnetic resonance cisternography
MRI	magnetic resonance imaging;
SNR	signal-to-noise ratio
TOF	time-of-flight
